# Successful Management of Rapidly Progressive Interstitial Pneumonia With Autoimmune Features in an Elderly Patient: A Case Report

**DOI:** 10.7759/cureus.78303

**Published:** 2025-01-31

**Authors:** Yudai Tanaka, Ryuichi Ohta, Chiaki Sano

**Affiliations:** 1 Commnity Care, Unnan City Hospital, Unnan, JPN; 2 Community Care, Unnan City Hospital, Unnan, JPN; 3 Community Medicine Management, Shimane University Faculty of Medicine, Izumo, JPN

**Keywords:** autoimmune diseases, cyclophosphamide, elderly patients, family medicine, general medicine, interstitial lung diseases, methylprednisolone, respiratory failure

## Abstract

An 82-year-old man presented with acute respiratory distress, a one-week history of dry cough, and worsening dyspnea. Chest computed tomography revealed bilateral diffuse ground-glass opacities, raising suspicion of rapidly progressive interstitial pneumonia. Rapid autoantibody testing confirmed interstitial pneumonia with autoimmune features (IPAF), likely triggered by an upper respiratory infection. Initial treatment with high-dose steroid pulse therapy was insufficient to stabilize the patient’s respiratory status. Cyclophosphamide pulse therapy was initiated on day 4, resulting in significant improvement by day 7. The patient’s oxygen requirements steadily decreased, and follow-up imaging showed near-complete resolution of lung abnormalities. Intensive immunosuppressive therapy, infection control measures, and tailored supportive care enabled functional recovery and discharge to a rehabilitation facility. This case highlights the importance of early diagnosis, rapid immunological evaluation, and aggressive immunosuppressive therapy in managing rapidly progressive interstitial pneumonia in elderly patients. Individualized treatment plans based on overall health rather than biological age can significantly improve outcomes, even in critically ill elderly patients. Early initiation of multidisciplinary care is crucial to achieving remission and restoring quality of life in this challenging population.

## Introduction

Acute rapidly progressive interstitial pneumonia is often a fatal condition, particularly in elderly patients, where the mortality rate exceeds 50%, posing significant treatment challenges [1]. Among the various subtypes, cases presenting with diffuse alveolar damage tend to deteriorate over days, emphasizing the critical importance of early diagnosis and prompt initiation of treatment [2]. However, there is currently insufficient academic validation regarding the optimal diagnostic workup and timing of treatment initiation [3].

Therapeutic intensity often sparks debate in elderly patients due to immune status variations and organ dysfunction [4]. In contemporary geriatric medicine, treatment decisions are increasingly based on biological age, the patient’s overall health status, and the severity of comorbidities [5]. This principle should also apply to acute, rapidly progressive interstitial pneumonia. On the other hand, in aging populations, particularly in remote and rural areas, treatment plans often tend toward undertreatment.

In this report, we present a case of acute, rapidly progressive interstitial pneumonia in a super-elderly patient. An early, rapid differential diagnosis and aggressive treatment approach successfully induced remission and restored the patient’s activities of daily living (ADL). Through this case, we discuss the necessity of accurately assessing pre-morbid conditions and providing appropriate intensive care tailored to elderly patients with acute interstitial pneumonia.

## Case presentation

An 82-year-old man presented to a local hospital with a chief complaint of difficulty breathing. One week before his visit, he began experiencing a dry cough. Two days before admission, his difficulty breathing worsened, accompanied by coughing, nasal discharge, and a low-grade fever. On the day of presentation, his symptoms, including difficulty breathing and fatigue, exacerbated to the point of impairing mobility, prompting him to visit his primary care physician. Upon evaluation, his oxygen saturation (SpO₂) was in the 60% range, and bilateral lung crackles were detected. A chest X-ray revealed bilateral infiltrates, leading to his emergency transfer to our hospital.

The patient’s past medical history included bronchial asthma, hypertension, dyslipidemia, osteoporosis, intraductal papillary mucinous neoplasm (IPMN), and chronic subdural hematoma. His medications included telmisartan, levocetirizine, eldecalcitol, magnesium oxide, and inhaled budesonide/formoterol.

On arrival, his vital signs were as follows: clear consciousness, blood pressure 130/66 millimeters of mercury (mmHg), pulse rate 90 beats per minute (bpm), body temperature 38.5 degrees Celsius, respiratory rate 30 breaths per minute (bpm), and SpO₂ 88-90% on an 8-liter oxygen mask. Physical examination in the sitting position showed no jugular vein distension or signs of labored breathing. Bilateral crackles were audible throughout the lungs without wheezing. Peripheral extremities were warm, with no heart murmurs or leg edema. No remarkable findings were observed on the face, fingers, skin, joints, eyes, or abdomen.

Laboratory tests showed normal serum Krebs von den Lungen-6 (KL-6) levels but elevated lactate dehydrogenase (LDH), surfactant protein D (SP-D), and surfactant protein A (SP-A) (Table 1).

**Table 1:** Initial laboratory data of the patient CRP, C-reactive protein; C3/4, Complement 3/4; KL-6, Krebs von den Lungen-6; SP-A/D, surfactant protein A/D; anti-ARS, anti-aminoacyl-tRNA synthetase; anti-MDA5, anti-melanoma differentiation-associated gene 5; MPO-ANCA, anti-myeloperoxidase antineutrophil cytoplasmic antibody

Parameter	Level	Reference
White blood cells	9.9	3.5–9.1 × 10^3^/μL
Neutrophils	82.8	44.0–72.0%
Lymphocytes	8.0	18.0–59.0%
Hemoglobin	10.2	11.3–15.2 g/dL
Hematocrit	30.7	33.4–44.9%
Mean corpuscular volume	97.6	79.0–100.0 fl
Platelets	34.3	13.0–36.9 × 10^4^/μL
Total protein	6.1	6.5–8.3 g/dL
Albumin	2.2	3.8–5.3 g/dL
Total bilirubin	1.0	0.2–1.2 mg/dL
Aspartate aminotransferase	80	8–38 IU/L
Alanine aminotransferase	80	4–43 IU/L
Alkaline phosphatase	388	38-113 U/L
γ-Glutamyl transpeptidase	300	16-73 IU/L
Lactate dehydrogenase	379	121–245 U/L
Blood urea nitrogen	15.9	8–20 mg/dL
Creatinine	0.90	0.40–1.10 mg/dL
Serum Na	137	135–150 mEq/L
Serum K	3.9	3.5–5.3 mEq/L
Serum Cl	102	98–110 mEq/L
CRP	11.3	<0.30 mg/dL
C3	67	86-160 mg/dL
C4	10	17-45 mg/dL
KL-6	284	105-401 U/mL
SP-D	127	<127 ng/mL
SP-A	131.3	<43.8 ng/ml
Antinuclear antibody level	<40	<40
Cytoplasm	40	<40
Anti-SSA antibody	<1.0	<1.0
Anti-CCP antibody	<0.6	<0.6
Anti-ARS antibody	<5.0	<5.0
Anti-MDA5 antibody	<4	<4
MPO-ANCA	<1.0	<1.0 U/mL
β-D-glucan	16.1	<20 pg/m
Urine test	-	-
Leukocyte	Negative	Negative
Protein	Negative	Negative
Blood	Negative	Negative

**Figure 1:**
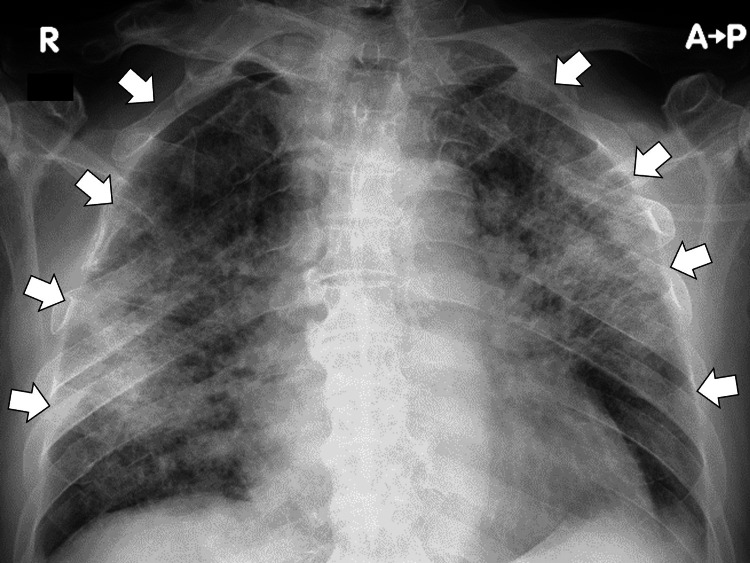
Chest X-ray revealing bilateral infiltrates (white arrows)

A chest X-ray revealed bilateral infiltrates (Figure 1).

**Figure 2:**
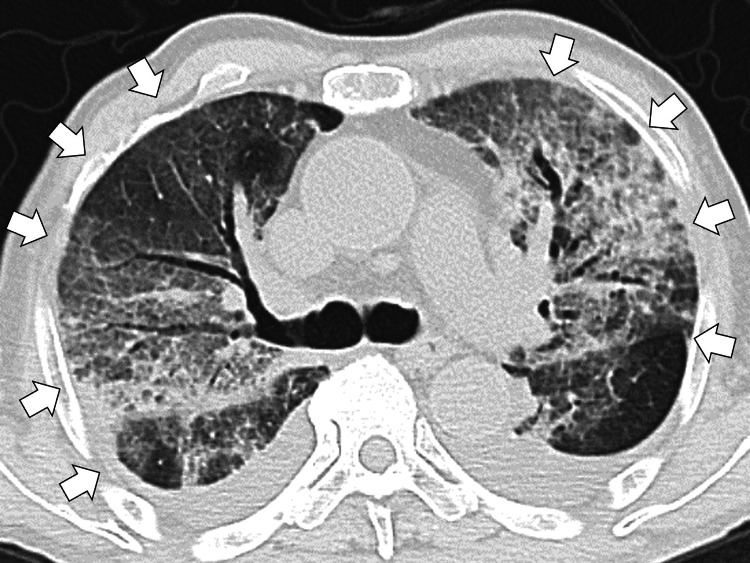
Computed tomography of the chest showing diffuse ground-glass opacities in both lungs (white arrows)

Computed tomography (CT) of the chest showed diffuse ground-glass opacities in both lungs (Figure 2).

Urinary antigen tests for *Streptococcus pneumoniae* and *Legionella* were negative, as were sputum and pharyngeal swab antigen tests for *Mycoplasma* and *Chlamydia*. Given the absence of significant sputum production and the predominant interstitial shadowing on CT, acute rapidly progressive interstitial pneumonia was suspected.

Bacterial pneumonia could not be excluded on the day of admission, and ceftriaxone (2 grams per day) was administered intravenously. Concurrently, methylprednisolone (1,000 milligrams per day) was given for three days, followed by prednisolone at 1 milligram per kilogram. On day 4, oxygen demand increased, requiring high-flow nasal cannulation (HFNC) with a fraction of inspired oxygen (FiO₂) of 90%. Autoantibody testing revealed cytoplasmic antineutrophil cytoplasmic antibodies (c-ANCA) at a titer of 1:80, while anti-aminoacyl-tRNA synthetase (anti-ARS) and anti-melanoma differentiation-associated gene 5 (anti-MDA5) antibodies were negative. Based on clinical findings, the patient was diagnosed with interstitial pneumonia with autoimmune features (IPAF), likely triggered by an upper respiratory infection.

**Figure 3:**
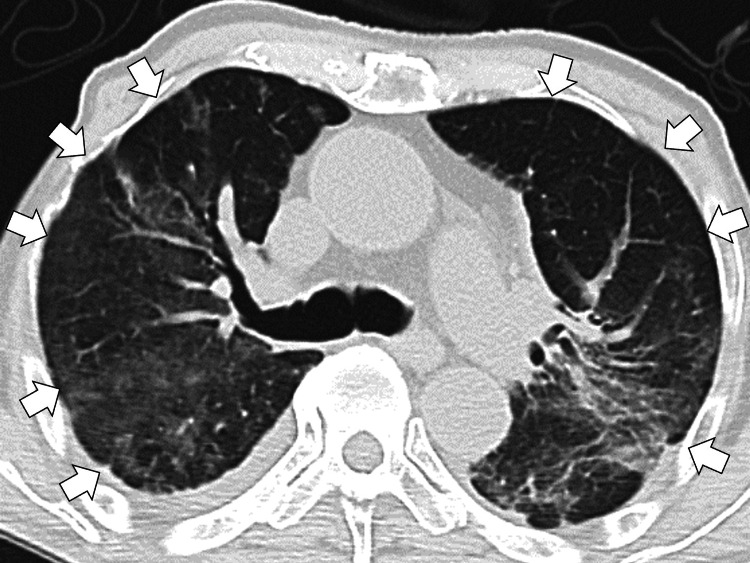
Follow-up chest computed tomography revealing near-complete resolution of ground-glass opacities (white arrows)

Given the poor response to steroid pulse therapy and the possibility of refractory interstitial pneumonia with autoimmune features, cyclophosphamide pulse therapy (500 milligrams per day) was initiated on day 4. By day 7, his respiratory condition began to improve. By day 14, the fraction of inspired oxygen (FiO₂) was reduced to 30%, enabling the discontinuation of high-flow nasal cannulation and initiation of oxygen therapy via mask. On day 21, oxygen saturation was maintained in the 90% range with 1-liter per minute nasal cannula oxygen. Follow-up chest computed tomography revealed near-complete resolution of ground-glass opacities (Figure 3).

The patient was transferred to a rehabilitation ward on day 23 to begin intensive rehabilitation. Prednisolone was tapered biweekly, and tacrolimus (3 milligrams per day) was added. Ultimately, prednisolone was successfully discontinued.

## Discussion

This case represents a rare instance of successful interstitial pneumonia treatment with IPAF in an elderly patient triggered by an upper respiratory infection. Early diagnosis and the appropriate administration of high-intensity immunosuppressive therapy were critical in achieving a favorable outcome. Acute interstitial pneumonia in elderly patients has a high mortality rate, particularly in cases exhibiting diffuse alveolar damage (DAD), where rapid diagnosis and therapeutic intervention are essential.

The importance of early diagnosis and timely initiation of treatment in acute rapidly progressive interstitial pneumonia in elderly patients cannot be overstated. In such cases, prompt clinical evaluation and imaging studies significantly influence prognosis [6]. In this patient, initial symptoms of dry cough and difficulty breathing, combined with bilateral diffuse ground-glass opacities on imaging, strongly suggested IPAF. Rapid autoantibody testing and immunological evaluation facilitated timely disease identification and prevented treatment delays. Similarly, the previous study demonstrated the effectiveness of early diagnosis in elderly IPAF patients [7]. Despite this, the prognosis for elderly patients with IPAF remains poor. Early and aggressive treatment strategies should be considered to improve outcomes and maintain activities of daily living (ADL) [8].

Aggressive treatments such as steroid pulse therapy and cyclophosphamide appear to be effective in elderly patients with rapidly progressive interstitial pneumonia [9]. In this case, steroid pulse therapy (1,000 mg of methylprednisolone for three days) was implemented as an initial treatment but was insufficient to improve respiratory status. Consequently, early administration of cyclophosphamide pulse therapy (500 mg) likely suppresses inflammation and controls lung damage. The previous study reported that cyclophosphamide pulse therapy is particularly effective in the acute management of refractory IPAF [10]. Increasing evidence, including the present case, supports aggressive treatments for acute, rapidly progressive interstitial pneumonia in elderly patients [11]. However, further research on aggressive therapy for super-elderly patients with IPAF remains necessary.

In managing IPAF in elderly patients, it is essential to consider pre-admission ADL and tailor treatment intensity accordingly. Treatment intensity should be adjusted based on the patient’s overall health and biological age for acute interstitial pneumonia [12]. In this case, the patient’s relatively well-controlled comorbidities allowed for high-intensity immunosuppressive therapy, successfully enabling recovery from the acute phase and social reintegration. A previous study highlighted the importance of risk management and the efficacy of immunosuppressive treatment in elderly patients [13].

Aggressive treatment of acute rapidly progressive interstitial pneumonia necessitates careful differentiation from infectious diseases and management of complications. Differentiating infectious pneumonia from other causes of acute respiratory failure in elderly patients is critical. In this case, rapid bacterial cultures, urinary antigen tests, and autoantibody testing ruled out infectious pneumonia, allowing treatment for autoimmune pneumonia to commence [14,15]. Preventing secondary infections during treatment is also crucial, necessitating the appropriate use of antibiotics and careful immunosuppressive management [16]. Similar clinical reports emphasize the importance of secondary infection prevention for acute interstitial pneumonia intensive care [17]. While timely treatment initiation is paramount, avoiding immunosuppression that could exacerbate infectious diseases is equally critical. Thus, differential diagnosis should be as thorough as possible, based on clinical progression and initial test results. Future case reports and studies should investigate further the possibility of intensive care of IPAF among older patients and the balance between risks and benefits for them.

## Conclusions

In this case, early diagnosis and multidisciplinary treatment successfully saved the life of an elderly patient with IPAF. Specifically, the combination of steroid and cyclophosphamide pulse therapy contributed to disease control and functional recovery. For acute rapidly progressive interstitial pneumonia in elderly patients, treatment intensity should not be limited based solely on biological age. Instead, aggressive therapeutic interventions based on individualized clinical evaluation are necessary.

## References

[1] Toyoda Y, Hanibuchi M, Kishi J (2016). Clinical features and outcome of acute exacerbation of interstitial pneumonia associated with connective tissue disease. J Med Invest.

[2] Mukhopadhyay S, Parambil JG (2012). Acute interstitial pneumonia (AIP): relationship to Hamman-Rich syndrome, diffuse alveolar damage (DAD), and acute respiratory distress syndrome (ARDS). Semin Respir Crit Care Med.

[3] Churg A, Wright JL, Tazelaar HD (2011). Acute exacerbations of fibrotic interstitial lung disease. Histopathology.

[4] Kaarteenaho R, Kinnula VL (2011). Diffuse alveolar damage: a common phenomenon in progressive interstitial lung disorders. Pulm Med.

[5] Akashi Y, Horinishi Y, Sano C, Ohta R (2023). Deciding a treatment plan for an older patient with severe idiopathic pulmonary fibrosis: a case report. Cureus.

[6] Mikolasch TA, Garthwaite HS, Porter JC (2017). Update in diagnosis and management of interstitial lung disease. Clin Med (Lond).

[7] Patterson KC, Shah RJ, Porteous MK (2017). Interstitial lung disease in the elderly. Chest.

[8] Castriotta RJ, Eldadah BA, Foster WM (2010). Workshop on idiopathic pulmonary fibrosis in older adults. Chest.

[9] Vacchi C, Sebastiani M, Cassone G, Cerri S, Della Casa G, Salvarani C, Manfredi A (2020). Therapeutic options for the treatment of interstitial lung disease related to connective tissue diseases. A narrative review. J Clin Med.

[10] Wiertz IA, van Moorsel CH, Vorselaars AD, Quanjel MJ, Grutters JC (2018). Cyclophosphamide in steroid refractory unclassifiable idiopathic interstitial pneumonia and interstitial pneumonia with autoimmune features (IPAF). Eur Respir J.

[11] van den Bosch L, Luppi F, Ferrara G, Mura M (2022). Immunomodulatory treatment of interstitial lung disease. Ther Adv Respir Dis.

[12] Raghu G, Collard HR, Egan JJ (2011). An official ATS/ERS/JRS/ALAT statement: idiopathic pulmonary fibrosis: evidence-based guidelines for diagnosis and management. Am J Respir Crit Care Med.

[13] Borren NZ, Ananthakrishnan AN (2019). Safety of biologic therapy in older patients with immune-mediated diseases: a systematic review and meta-analysis. Clin Gastroenterol Hepatol.

[14] Oryoji K, Himeji D, Nagafuji K, Horiuchi T, Tsukamoto H, Gondo H, Harada M (2005). Successful treatment of rapidly progressive interstitial pneumonia with autologous peripheral blood stem cell transplantation in a patient with dermatomyositis. Clin Rheumatol.

[15] Rajan SK, Cottin V, Dhar R (2023). Progressive pulmonary fibrosis: an expert group consensus statement. Eur Respir J.

[16] Stevens DL, Bisno AL, Chambers HF (2005). Practice guidelines for the diagnosis and management of skin and soft-tissue infections. Clin Infect Dis.

[17] Hayat Syed MK, Bruck O, Kumar A, Surani S (2023). Acute exacerbation of interstitial lung disease in the intensive care unit: Principles of diagnostic evaluation and management. World J Crit Care Med.

